# Clients' reasons for prenatal ultrasonography in Ibadan, South West of Nigeria

**DOI:** 10.1186/1472-6874-9-12

**Published:** 2009-05-09

**Authors:** Christopher A Enakpene, Imran O Morhason-Bello, Anthony O Marinho, Babatunde O Adedokun, Adegoke O Kalejaiye, Kayode Sogo, Sikiru A Gbadamosi, Babatunde S Awoyinka, Obehi O Enabor

**Affiliations:** 1 Department of Obstetrics and Gynecology, William Beaumont Hospital, 3601 W, 13 Mile Road, Royal Oak, MI 48073, USA; 2 Department of Obstetrics & Gynaecology, University College Hospital, Ibadan, Oyo state, Nigeria; 3 St. Gregory's Specialist Clinic and Ultrasound Diagnostic Centre, Yemetu, Ibadan, Oyo state, Nigeria; 4 Department of Epidemiology, Medical Statistics, and Environmental, Health, College of Medicine, Ibadan, Oyo state, Nigeria

## Abstract

**Background:**

Prenatal ultrasonography has remained a universal tool but little is known especially from developing countries on clients' reasons for desiring it. Then aim was to determine the reasons why pregnant women will desire a prenatal ultrasound.

**Methods:**

It was a cross-sectional survey of consecutive 222 women at 2 different ultrasonography facilities in Ibadan, South-west Nigeria.

**Results:**

The mean age of the respondents was 30.1 ± 4.5 years. The commonest reason for requesting for prenatal ultrasound scans was to check for fetal viability in 144 women (64.7%) of the respondents, followed by fetal gender determination in 50 women (22.6%. Other reasons were to check for number of fetuses, fetal age and placental location. Factors such as younger age, artisans profession and low level of education significantly influenced the decision to check for fetal viability on bivariate analysis but all were not significant on multivariate analysis. Concerning fetal gender determination, older age, Christianity, occupation and gravidity were significant on bivariate analysis, however, only gravidity and occupation remained significant independent predictor on logistic regression model. Women with less than 3 previous pregnancies were about 4 times more likely to request for fetal sex determination than women with more than 3 previous pregnancies, (OR 3.8 95%CI 1.52 – 9.44). The professionals were 7 times more likely than the artisans to request to find out about their fetal sex, (OR 7.0 95%CI 1.47 – 333.20).

**Conclusion:**

This study shows that Nigerian pregnant women desired prenatal ultrasonography mostly for fetal viability, followed by fetal gender determination. These preferences were influenced by their biosocial variables.

## Background

Pregnancy is usually associated with high expectations and joy for most women, but to some, it is a journey to an unknown destination and it may be accompanied by vary degree of anxiety state [1,2]. The cause of this anxiety may be due to the concern of the expectant mothers about the viability of the pregnancy at all stages and it is especially worsen in first timers [2,3]. One of the strategies employed to reduce this anxiety state is prenatal ultrasound scan [4,5]. Sometimes, the result obtained from the scan may cause negative psychological effect when an undesired report is presented to the pregnant woman [6]. This has sometimes resulted in patients rejecting the result claiming spiritual powers to overcome evil (Marinho A.O. Personal Communication 2008).

Prenatal ultrasonography is one investigative tool that has revolutionized obstetric practice and its usefulness is expanding. Prenatal ultrasound scans are usually requested by health care providers as either part of routine baseline prenatal evaluation or for specific medical indications in the course of the pregnancy. In many settings, ultrasound scan is a prerequisite and part of the prenatal evaluation armamentaria [7,8]. Opinions are still divided in regards to the rationale for routine prenatal ultrasound scans in a normal population base on the fact that there are no convincing evidences of its benefit at the moment in reducing perinatal mortality [9-11]. Some regulatory bodies do not support the routine use of prenatal ultrasound in a low risk population [12]. Ultrasound is requested at different stages of pregnancy for indicated and routine assessment.

In early first trimester, ultrasound scans can be used for pregnancy confirmation, location, dating and determination of multiple pregnancies. In mid-trimester, routine ultrasonography is useful for prenatal diagnosis especially between 18 and 24 weeks to rule out any fetal anomaly. In late trimester, the benefits of ultrasonography include: determination of fetal lie, presentation and estimated weight, localization of placental, amniotic fluid volume estimation and to guide invasive procedures such as amniocentesis and fetal cord sampling and intra-uterine transfusion.

Most of the aforementioned routine indications are provider initiated and expectedly, the clients should be adequately informed about the reason for such request. The Sonologist before the procedures should reinforce this information. However, studies have shown that women are not optimally offered this expected service [13,14]. Evidences have shown that the views of pregnancy women about prenatal ultrasound is crucial and varied [5,15]. It ranges from their perception on safety, the benefit and limitations and their personal reasons for requesting prenatal scan [4]. However, pregnant women may have their own reasons for consenting to undergoing prenatal ultrasound scans. Various studies have reported that some women may initiate the request for ultrasonography for both medical and non-medical reasons such as to see the baby, to determine fetal sex and spousal reassurance about the pregnancy state [4]. Gudex et al had also demonstrated that the preference is influenced by socio-demographic, obstetric and attitudinal factors of women [16]. In Nigeria, the available few studies on this topic focused mainly on the pregnant women's view about prenatal sex determination by the Sonologist during scanning [17,18]. From the observation in our clinic, most pregnant women now come with the results of ultrasound scan at their first prenatal visits. However, no study has been conducted to explore the reasons why pregnant women may initiate the request to undergo ultrasound scan even before they present to the healthcare provider for their first prenatal visit.

There has been an increasing trend of prenatal ultrasound scan in Nigeria in the last three decades especially in the urban centers [19,20]. Moreover, there are reports about women who patronize traditional birth attendants and mission homes undergoing prenatal ultrasound scanning as part of their evaluations. Currently, there are no national policies guiding the proliferation of providers of this procedure as well as whether pregnant women should have routine prenatal scan or not. Little is also known about the reasons why pregnant women in Nigeria crave for prenatal ultrasound scan. In our practice, either during antenatal clinic or informally, pregnant women often express their desire to have ultrasound for personal reasons. It is on this basis that we decided to carry out this study to find out the reasons why Nigerian pregnant women make personal request to undergo prenatal ultrasound scan and also to identify factors that may influence such decisions. This is in anticipation that the findings of this study may stimulate policy makers in Nigeria to take a drastic stand in regards to policy formulation and implementation on the use of prenatal ultrasound scan.

## Methods

### Study population

The study assessed the reason(s) for requesting ultrasound scan among pregnant women in Ibadan. The subjects recruited were consecutive pregnant women that personally presented for prenatal scan at the antenatal clinic of the department of obstetrics and gynecology, University College Hospital, Ibadan and St. Gregory's Specialist Clinic and Ultrasound Diagnostic Center, Yemetu, Ibadan. The study was conducted from February to April 2007.

### Design

It was descriptive cross-sectional survey of consecutive pregnant women that presented on their own for prenatal ultrasound scan during the study period.

### Setting

This study was conducted in two centers, University College Hospital, Ibadan – a tertiary center and St. Gregory's specialist clinic, Yemetu, Ibadan – a secondary center. The University College Hospital, Ibadan is a tertiary hospital that serves as referral center for private and public primary and secondary health facilities within Nigeria. The University College Hospital was initially commissioned with 500 bed spaces in 1957, but presently, the hospital has 850 beds and 163 examination couches. The current bed occupancy ranges from 75% to 90%. St. Gregory specialist clinic, Yemetu is a secondary health center that does predominantly diagnostic ultrasound scans. An average of 100 pregnant women are seen daily at this institution. The proposal for this study was approved by the joint commission of the College of Medicine, University of Ibadan/University College Hospital institutional review committee as part of partial fulfillment of the fellowship of West African College of Surgeon, part II examination.

### Data collection

Pretested structured questionnaires were administered to 222 consenting pregnant women who presented for prenatal ultrasound scan at the study centers on their own volition. The resident doctors who were the interviewers guided each of the respondents to fill-in the required information.

All subjects were interviewed about their socio-demographic characteristics, past obstetric history, history of index pregnancy, last menstrual period and reason(s) for requesting the prenatal ultrasound scan.

### Statistical analysis

The data obtained were coded and entered into SPSS software (14.5 versions, Chicago, Illinois). Bivariate analysis was performed using Chi-square test for categorical variables. Multivariate analysis was performed using logistic regression model to identify likely biosocial predictors such as age, ethnic group, religion, occupation and education on the two most common reasons for requesting prenatal ultrasound, which were fetal viability and gender determination. Fetal survival in this study encompasses fetal well-being, fetal anomaly and the likelihood of neonatal survival. The level of statistical significance was set at 0.05 and at 95 percent confidence level.

## Results

Two hundred and twenty two women were interviewed with a mean age of 30.1 years (SD = 4.5). Majority of the subjects, 87 (39.2%) were artisans, this was followed by 53 (23.9%) who were civil servants. Professionals constituted 18.5% while other professions made up of the remaining 18.4%. The proportion of women with tertiary education was 61.7%, while the rest were women with secondary education or less. There were 86.2% who were of the local Yoruba ethnic group. Almost all the women were married (97.3%) and 74.7% were Christians and the rest were Muslims (table 1). The mean gestational age of the women was 24 weeks (SD = 9.4), about 50% of the respondents were in the third trimester. The median number of pregnancies by the respondents was 2. The proportion of women who were having ultrasound scan for the first time in their life was 13.5%. However 38.3% of the women were having their first scan in the current pregnancy.

**Table 1:** Socio-demographic characteristics (n = 222)

**Variable**	**Percentage**
**Age (years)**	
<25	7.7
25–29	34.2
30–34	41.9
35+	16.2
**Ethnic group**	
Yoruba	86.2
Others	13.8
**Religion**	
Christianity	74.7
Islam	25.3
**Occupation**	
Artisans	39.2
Civil servants	23.9
Professionals	18.5
Others	18.4
**Education**	
Primary	9.1
Secondary	29.2
Tertiary	61.7
**Gravidity**	
One	21.5
Two	31.4
Three	21.5
Four or more	25.7
**Gestational age (Trimester)**	
First	17.6
Second	34.4
Third	48.0

**Figure 1:**
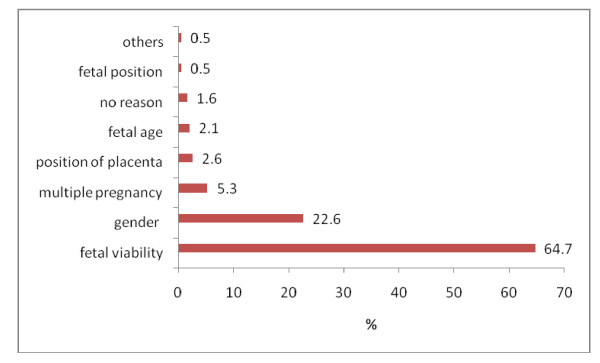
**What women desire to know during prenatal ultrasonography from the Sonologists**.

The commonest reason these women presented for prenatal ultrasound scan was to check fetal viability in 144 women (64.7%). This was followed by desires to know their baby's gender in 50 women (22.6%). Other reasons such as to find out if they were carrying multi-fetal pregnancies was found among 12 women (5.3%). Five women (2.1%) wanted to know about the gestational age of their fetuses and 1 woman (0.5%) requested for prenatal ultrasound scan to find out about the position of the placenta (Figure 1).

The relationship between reasons for prenatal ultrasound scans; categorised as fetal viability and others, and patient's variables are shown in table 2. Women younger than 30 years were more likely to request for fetal viability during prenatal ultrasound scan compare with women who were 30 years old or less. The request for fetal viability was also found to be inversely proportional to the women's chronological age at a statistically significant level (p = 0.003). There were more artisans 180 (81.1%) requesting for fetal viability as compared to other occupational groups and this was at a statistically significant level (p < 0.001). Moreover, patients with lower educational status were more likely to request fetal viability as compared with those of higher educational status at a statistically significant level (p = 0.033). Religion, ethnic group, gravidity and sex of previous babies were not found to significantly influence the women's reasons for requesting for prenatal ultrasound scan. Further analysis using multivariate analysis did not show any association between fetal viability as reason for scan request and these variables.

**Table 2:** Relationship between reasons for scan considering fetal viability versus other reasons and variables

**Variable**	**Fetal viability** **(% within the category)**	p value*
**Age (years)**		
<30	75.3	0.003*
30–34	63.5	
35+	42.9	
**Religion**		
Christianity	61.8	0.091
Islam	75.8	
**Occupation**		
Artisans	81.1	<0.001*
Civil servants	54.3	
Professionals	42.9	
Other	65.7	
**Education**		
Secondary or less	75.0	0.033*
Tertiary	59.6	
**Ethnic group**		
Yoruba	63.3	0.157
Others	78.3	
**Gravidity**		
1	70.3	0.794
2	61.8	
3	61.8	
4+	60.4	
**Sex of previous babies**		
All males	64.2	0.086
All females	50.0	
Mixed	64.9	
Nulliparous	81.8	
**Gestational age ***(Trimester)*		
First	82.4	0.475
Second	66.7	
Third	68.6	

* statistically significant at P < 0.05

Table 3 shows cross-tabulations between desires to know fetal gender as a reason for prenatal ultrasound scans as compared with other reasons. There is a statistical significant relationship between the ages of the women and desire to know fetal gender (p = 0.001). The desire to know the fetal gender was directly related to the chronological age of the respondents. In addition, the religion of the women was also found to have significant association with desire for the women to know the fetal gender (p = 0.004). The Christians were more likely to request for their fetal gender than the Muslim, but not at a statistical significant level (p = 0.085). Occupation was also found to significantly influence the reasons to know fetal gender (p = 0.002), artisans were less keen to know about their fetal gender as compared with other professions. The number of previous pregnancies and deliveries of the women were statistically significant (p = 0.007), as the desire to know fetal gender increases with higher parity as compared with lower parity. Age, education, tribe and sex of previous babies were not found to have statistical significant influence on the women's desires to know the fetal gender.

**Table 3:** Relationship between reasons for scan considering gender versus other reasons and some variables

**Variable**	**Gender** **(% within the category)**	p value*
**Age (years)**		
<30	13.6	0.001*
30–34	21.6	
35+	45.7	
**Religion**		
Christianity	27.1	0.004*
Islam	6.7	
**Occupation**		
Artisans	8.1	0.002*
Civil servants	32.6	
Professionals	31.4	
Other	31.4	
**Education**		
Secondary or less	15.3	0.072
Tertiary	26.6	
**Ethnic group**		
Yoruba	22.9	0.552
Others	17.4	
**Gravidity**		
1	10.8	0.007*
2	14.5	
3	35.3	
4+	35.4	
**Sex of previous babies**		
All males	26.4	0.191
All females	34.1	
Mixed	27.0	
Nulliparous	9.1	
**Gestational age ***(Trimesters)*		
First	0.0	0.108
Second	15.4	
Third	21.6	

* statistically significant at P < 0.05

Further analysis using logistic regression model to identify independent predictors of reasons pregnant women desire to know fetal gender shows that only gravidity and occupational status retain their association. Women with three or less than three previous pregnancies were more likely to request to know fetal sex than those who have had more than three previous pregnancies, OR: 3.8 (95% CI = 1.52 – 9.44). This is especially true if any of the previous pregnancies did not result in a live birth. In addition, Professional women were also more likely to request to know their fetal sex than artisans, OR: 7.0 (95% CI OR = 1.47–333.2) (Table 4).

**Table 4:** Logistic regression of reasons for scan (gender versus other reasons) on patient's characteristics, + statistically significant

**Variable**	**β**	**Odds ratio**	**p value**	**95% CI OR**
**Education**				
Secondary or less versus tertiary	0.298	1.35	0.640	0.21–2.59
**Age (years)**				
<30 versus 30+	0.199	1.22	0.708	0.43–3.46
**Gravidity**				
<3 versus 3+	1.332	3.79	0.004^+^	1.52–9.44
**Religion**				
Islam versus Christianity	1.209	3.36	0.085	0.85–13.33
**Occupation**				
Professionals versus artisans	1.945	7.00	0.014^+^	1.47–333.20
Professionals versus Civil servants	0.164	1.18	0.776	0.382–3.632
Professionals versus Others	0.602	1.825	0.371	0.488–6.825

## Discussion

The clients' perception of any investigational tool may influence their belief and subsequent compliance. This study highlights reasons why pregnant Nigerian women would want to have prenatal ultrasound scan on their own volition. It has been observed especially from developing countries that women are often neglected on issues that bother on their health [21]. Despite the high patronage of pregnant women at the ultrasound diagnostic centres in Nigeria, the authors are not aware of any study in the country that has investigated the reasons why pregnant women would want to perform obstetric ultrasound scan without being prompted.

Although, the population studied reflected a predominant Yoruba ethnic group and majority had tertiary education, however, socio-cultural settings could still have impact on some reproductive health decisions. Therefore, it is not unlikely that the views expressed by these women despite their educational background might have been influenced by their socio-cultural beliefs or community values.

In this study, the majority of the women that presented for prenatal ultrasound scan preferred to determine the viability of their fetuses, followed by sex determination. This may be due to the general anxiety that has been observed among pregnant women in many settings irrespective of their educational status, parity and race [2,3]. It stands to reason why a woman and her immediate family members eagerly look forward to pregnancy confirmation after her missed menstrual periods in a community where matrimonial success is hinged on conception and eventual delivery of a live baby. Currently, there is no national protocol for prenatal screening programme in Nigeria; the request is often on the initiative of the attending healthcare provider. Considering the high proportion of women that wanted fetal viability determined, it behooves policy makers and other related experts to explore ways of integrating prenatal ultrasound scans into our practice.

Biosocial variables such as age, occupation and educational attainment of respondents significantly influenced their choice of requesting fetal viability during prenatal ultrasound scan on bivariate analysis, but not on logistic regression analysis. Women of younger age group, artisans and of lower educational status were more likely to make fetal viability request. This observation may be due to the fact most women below 30 years may be carrying their first pregnancy. Hence, they are often more anxious to know about the viability of their pregnancies. Moreover, it is probably that younger aged women are more likely to be of lower gravity and also the artisan may not have the confidence of self-reassurance until there is ultrasound evidence that her pregnancy is normal. Women of lower educational and occupational status – artisans form the majority of those that patronise traditional birth attendants and mission homes in Nigeria. These facilities lack credible tool for assessing fetal viability. Hence, these groups of women often patronise private ultrasound centres to allay their anxiety. Although other factors such as gravidity and sex of previous baby do not significantly influence this decision, it is worth noting that the lower the gravidity of respondents, the more likely the probability for requesting ultrasound scan for confirming fetal viability. The sex of previous child does not have any significant influence on the decision to determine fetal viability in this study.

Antenatal sex determination has received attention in both developed and developing countries for various reasons. In some countries, there are strict laws barring this request to combat sex selection and fetocide. In other settings, fetal sex disclosure is regarded as a human rights issue that should not be denied upon request. In Nigeria, there are no laws guiding prenatal fetal sex request and determination. Significant numbers of pregnant Nigerian women are interested to know the sex of their unborn baby. This study showed that 23 percent of the women requested to know the gender of their unborn child during prenatal ultrasound scan. This observation is in consonant with another study by Okonta et al in Mid-western Nigeria [18].

Age, religion, occupation and gravidity significantly influenced decision of women in this study for sex determination on bivariate analysis. Among these factors, only gravidity and occupation status still remain significant after multivariate analysis. Women with less than three previous pregnancies were about 4 times more likely request to know their fetal gender than those with more than three previous pregnancies Similarly, professional women were 7 times more likely make a request to know about their fetal gender as compared with the artisans. However, the large width of this confidence interval for the odd ratio indicates a less reliable estimate and the result should be treated with caution. The identified factors in this study were similar to those reported by other studies in Nigeria [17]. Women who have smaller family sizes or desire to have small families are more under pressure for gender balancing; as such they may be more desperate to know about the gender of the unborn child. Some women end up with large family sizes due to their desperate desire for a particular gender. It therefore means that certain groups of women within Nigeria would want to know the sex of their baby during the prenatal period.

Other reasons mentioned by the respondents for prenatal ultrasound scans were determination of number of fetuses and fetal age, confirmation of placenta position and fetal presentation. However, some women had no specific reasons, but just wanted to see their fetus in-utero. However, further exploration of these reasons was not pursued due to smaller sample size on each subset. It would also have been interesting to determine the proportions of women that preferred each of these requests by trimesters as it may provide further insight.

## Conclusion

This study shows that pregnant Nigerian women desire prenatal ultrasonography mostly for fetal viability, followed by gender determination. Their preferences for prenatal ultrasonography were mostly influenced by their biosocial variables. It is therefore recommended that adequate information on the appropriate timing of prenatal ultrasonography, its role in obstetric care and its limitations should be provided for these women in order to achieve the optimal benefit of pregnancy outcome.

## Competing interests

The authors declare that they have no competing interests.

## Authors' contributions

CAE coordinated the design, data collection and writing of the manuscript as well as performed part of the prenatal ultrasound scan for participants; IOMB performed the literature search, participated in design and data collection as well as coordinated the writing of the manuscript. AM, AOK and SAG performed part of the scan and data collection. BOA performed the analysis and was also involved in the writing of the manuscript. BSA and OOE were involved in participant recruitment and data collection. All authors read and approved the final manuscript.

## Pre-publication history

The pre-publication history for this paper can be accessed here:


